# The draft genome of the blood pheasant (*Ithaginis cruentus*): Phylogeny and high‐altitude adaptation

**DOI:** 10.1002/ece3.6782

**Published:** 2020-09-28

**Authors:** Chuang Zhou, Yi Liu, Lu Qiao, Yang Liu, Nan Yang, Yang Meng, Bisong Yue

**Affiliations:** ^1^ Key Laboratory of Bioresources and Ecoenvironment (Ministry of Education) College of Life Sciences Sichuan University Chengdu China; ^2^ Chengdu Zoo/Chengdu Wildlife Research Institute Chengdu China; ^3^ Institute of Qinghai‐Tibetan Plateau Southwest Minzu University Chengdu China

**Keywords:** blood pheasant, comparative genomics, high‐altitude adaptation, missense mutation, phylogenetic analysis, positive selection

## Abstract

The blood pheasant (*Ithaginis cruentus*), the only species in the genus *Ithaginis*, lives in an extremely inhospitable high‐altitude environment, coping with hypoxia and ultraviolet (UV) radiation. To further investigate the phylogeny of Phasianidae species based on complete genomes and understand the molecular genetic mechanisms of the high‐altitude adaptation of the blood pheasant, we de novo assembled and annotated the complete genome of the blood pheasant. The blood pheasant genome size is 1.04 Gb with scaffold N50 of 10.88 Mb. We identified 109.92 Mb (10.62%) repetitive elements, 279,037 perfect microsatellites, and 17,209 protein‐coding genes. The phylogenetic tree of Phasianidae based on whole genomes revealed three highly supported major clades with the blood pheasant included in the “erectile clade.” Comparative genomics analysis showed that many genes were positively selected in the blood pheasant, which was associated with response to hypoxia and/or UV radiation. More importantly, among these positively selected genes (PSGs) which were related to high‐altitude adaptation, sixteen PSGs had blood pheasant‐specific missense mutations. Our data and analysis lay solid foundation to the study of Phasianidae phylogeny and provided new insights into the potential adaptation mechanisms to the high altitude employed by the blood pheasant.

## Graphical abstract

We de novo assembled and annotated the complete genome of the blood pheasant which is the only species in the genus Ithaginis. We conducted phylogenetic analysis of 20 Galliformes species based on whole genomes. The molecular genetic mechanisms of high‐altitude adaptation of the blood pheasant were investigated.

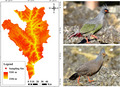

## 1 BACKGROUND

**FIGURE 1:**
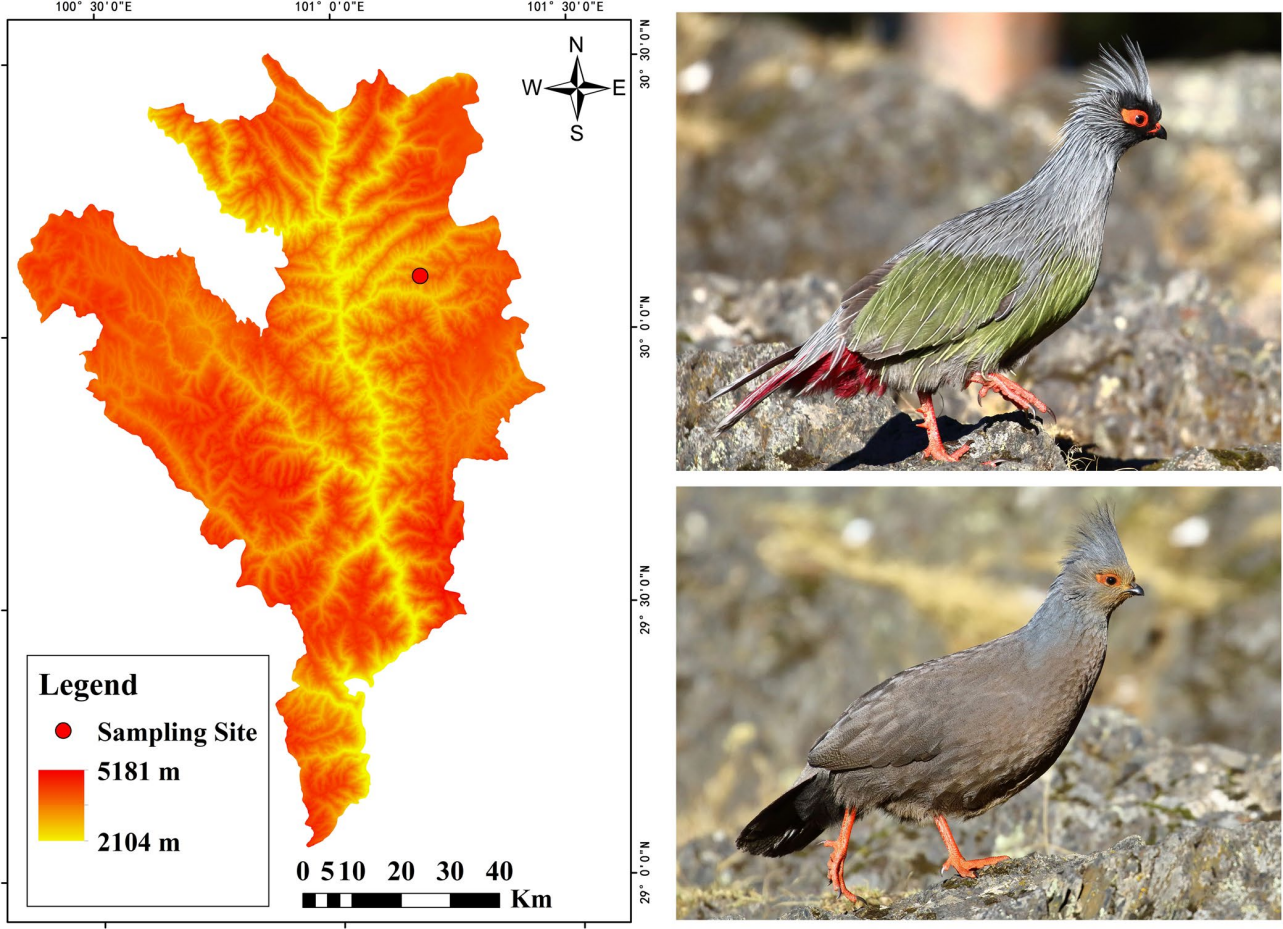
Sampling site and photographs of blood pheasant

The blood pheasant (*Ithaginis cruentus*, Phasianidae, Galliformes) is the only species in the genus *Ithaginis* (Zeng et al., 2013). This species is a monogamous galliform and widespread in eastern Himalayas, including China (Cheng, 1978). Blood pheasant prefers coniferous or mixed forests and scrub areas at altitudes of 3200–4700 m above sea level (Figure 1) (MacKinnon, Phillipps, & He, 2000). Although it has been evaluated as Least Concern by IUCN (IUCN 2012), the blood pheasant has been listed as the Category II of the Chinese protected animals legally, and its population has been reported to decrease slowly (Zeng et al., 2013). Currently, the genomic resources of the blood pheasant are very limited; the biology and ecology of this species remained largely unexplored; and more genetic information associated with this species is needed for further research.

Geographic barriers, topographic features, typically low temperatures, hypoxia, and strong ultraviolet radiation have influenced species differentiation and high‐altitude adaptability of the blood pheasant. The adaptation to high‐altitude environment can be attributed to advantageous genetic mutations and selective pressure. Many studies have been conducted to investigate the molecular genetic mechanisms underlying organisms’ high‐altitude adaptation in mammals like yak (*Bos grunniens*) (Qiu et al., 2012) and Tibetan antelope (*Pantholops hodgsonii*) (Ge et al., 2013). Among the responsible genes proposed by previous studies, the most prominent ones were *EPAS1* and *EGLN1* which were reported to be the key genes functioning at the upstream of the hypoxia‐inducible factor (HIF) pathway (Gorkhali et al., 2016). Convergent evolution was proved to shape the similar genes in the high‐altitude adaptation of different animals like *EPAS1* which was shared by Tibetans, Tibetan mastiff (*Canis lupus familiaris*), Tibetan grey wolf (*Canis lupus chanco*), and Tibetan goat (*Capra hircus*) (Gorkhali et al., 2016). However, species with different genetic background could obtain unique adaptive mechanisms like Tibetan pig (from Tibet, Gansu, Sichuan, and Yunnan provinces in China) (Ai et al., 2014). However, the genetic mechanism of high‐altitude adaptation in birds was poorly investigated (e.g., Tibetan chickens (Wang et al., 2015)). It is possible that the blood pheasant has adapted to high‐altitude conditions through different genes or functional pathways.

Besides comparative genomics approach, understanding themechanisms of adaptation to high‐altitude requires a robust phylogeny. Rapid radiation and convergent morphological evolution often result in conflicting phylogenetic relationships of species in the Phasianidae, one of four families in the Galliformes (Shen et al., 2014). Although many studies on the phylogenetic research have been conducted in the Phasianidae (Shen et al., 2014; Wang, Kimball, Braun, Liang, & Zhang, 2013), not surprisingly, many unsolved nodes and conflicts remain. The incongruence of phylogenetic relationships requires a reassessment of the phylogeny of the Phasianidae. Most previous phylogenetic analyses were performed based on either mitochondrial (mt) genes (Pereira & Baker, 2006; Shen et al., 2010), a few nuclear genes (Armstrong, Braun, & Kimball, 2001), or a combination of mt and a few nuclear gene sequences (Crowe et al., 2006; Kimball & Braun, 2008). Furthermore, mtDNA only reflect the matrilineal genealogy and not provide information on paternal contributions. With the fast development and reducing cost of next‐generation sequencing, an increasing number of Phasianidae species were sequenced (Cui et al., 2019; Zhou et al., 2018, 2019, 2020), which served as a powerful tool to infer phylogenetic relationships in the Phasianidae.

So far, the genomic resources of the blood pheasant are very limited for deeper bioinformatic analysis. In this study, we aimed to describe the first blood pheasant genome and to provide pivotal molecular material for the study of the blood pheasant and Phasianidae family. With availability and ease to produce HTS data (whole genome level), we performed phylogenetic comparative genomics analyses in order to increase our knowledge of the blood pheasant's high‐altitude adaptation and evolutionary history of Phasianidae species.

## 2 MATERIALS AND METHODS

### 2.1 Sampling and sequencing

Genomic DNA of blood pheasant was collected from muscle sample of a wild male in the museum of Sichuan University. DNA was isolated from the muscle using the Qiagen DNeasy Blood and Tissue Kit (Qiagen) following the manufacturer's protocol. Then, we constructed two kinds of libraries on Illumina HiSeq 2000 platform to sequence the genome using the whole genome shotgun approach, including two paired‐end sequence libraries with insert sizes of 230 base pairs (bp) and 500 bp, and three mate‐paired libraries with insert sizes of 2, 5, and 10 kb. Quality control composed of adaptor removal using Trimmomatic (Lohse et al., 2012), discard of short reads, trim of poor‐quality bases, and removal of all identical paired‐end reads (Doyle et al., 2014).

### 2.2 Genome assembly and completeness assessment

The contig and scaffold assemblies of the blood pheasant genome were carried out by the SOAPdenovo2 (Luo et al., 2012). Then, the obtained scaffolds were assembled into super‐scaffolds by SSPACE (Boetzer, Henkel, Jansen, Butler, & Pirovano, 2010). Finally, we used short‐insert reads from Illumina to correct the remaining intra‐scaffold gaps by Gapcloser. The completeness of genome assembly was assessed by Benchmarking Universal Single‐Copy Orthologs (BUSCO) analysis with the aves_odb9 database (Simão, Waterhouse, Ioannidis, Kriventseva, & Zdobnov, 2015).

### 2.3 Gene prediction and annotation

Two methodologies, homologous comparison and ab initio prediction, were used to annotate the protein‐coding genes (PCGs) in the blood pheasant. For ab initio prediction, we simultaneously employed two tools of AUGUSTUS (Stanke et al., 2006) and GENSCAN (Burge & Karlin, 1997), in which repetitive sequences were masked as "N" based on the HMM algorithm. For homologous annotation, protein sequences including red junglefowl (*Gallus gallus*), turkey (*Meleagris gallopavo*), zebra finch (*Taeniopygia guttata*), peregrine falcon (*Falco peregrinus*), and human (*Homo sapiens*) were aligned against the blood pheasant genome using TblastN (Altschul et al., 1997) with an E‐value cutoff of 1E‐5. The TblastN hits were then conjoined by SOLAR (Ashburner et al., 2000) to obtain the best matches. GeneWise (Birney, Clamp, & Durbin, 2004) was used to predict the exact gene structure of the corresponding genomic region on each TblastN hits. According to these two approaches, all the gene models were finally integrated by EVidenceModeler (EVM) (Haas et al., 2008). Repeatmasker (Smit, Hubley, & Green, 2010) was used to detect and classify different types of repetitive sequences, via aligning the genome sequences against the Repbase library (version 17.01). To further evaluate the repetitive DNA content within the blood pheasant genome, we utilized Krait (Du, Zhang, Liu, Zhang, & Yue, 2018) to detect and characterize genome‐wide tandem repeats (microsatellite loci), which could be used for identifying loci that could be employed in population genetic studies.

### 2.4 Functional annotation

We functionally annotated the predicted proteins within blood pheasant according to homologous searches against SwissProt and TrEMBL protein databases (Boeckmann et al., 2003) using BLASTP tools with the E‐value cutoff of 1E‐5. Gene Ontology (GO) descriptions of predicted genes were retrieved from SwissProt results. All genes were uploaded to KAAS (Moriya, Itoh, Okuda, Yoshizawa, & Kanehisa, 2007) in order to find the best matching and involvement pathway for each gene. This is a web server for annotating genetic functions of the artificially modified KEGG gene database by BLAST using the bidirectional click (BBH) method.

### 2.5 Analyses of gene family, phylogeny, and divergence

Gene families were constructed according to orthoMCL (Li, Stoeckert, & Roos, 2003). The detailed information of genomes used in this study was shown in Table S1. Phylogenetic analyses of these nine birds were constructed using 1:1 orthologous genes. Coding sequences from each 1:1 orthologous family were aligned by PRANK (Löytynoja & Goldman, 2010) and concatenated to one sequence for each species for building the tree. Then, the phylogenetic analysis was performed using maximum‐likelihood (ML) algorithm in RAxML (Stamatakis, 2014). The best‐scoring ML tree was inferred by rapid BP algorithm and ML searches after performing 1,000 rapid bootstraps. In this process, we implemented the Monte Carlo Markov Chain algorithm for divergence time estimation by MCMC tree tool in PAML package (Yang, 2007).

### 2.6 Positive selection analysis

Nine birds (blood pheasant, turkey, red junglefowl, Hainan partridge (*Arborophila ardens*), peregrine falcon, mallard (*Anas platyrhynchos*), rock dove (*Columba livia*), zebra finch, ostrich (*Struthio camelus*)) were chosen for positive selection analysis. For each gene, the branch‐site model in CODEML program from PAML package (Yang, 2007) was employed to detect whether the gene received positive selection in the blood pheasant branch. Two models were conducted to test the statistical significance of selective pressure specifically on the blood pheasant branch. One was the one‐ratio model acting as the null model (NSsites = 0, model = 0), and the other was model 2 (NSsites = 2). The two models were compared with the likelihood ratio test (LRT), which was calculated from the log likelihood (lnL) values for both models. The *p* values were obtained by calculating twice the difference between lnL (model2) and lnL (one‐ratio) and compared with a chi‐square distribution. We then identified positively selected genes (PSGs) of the blood pheasant by means of FDR adjustment with Q values < 0.05. GO enrichment analysis of PSGs was implemented by the KOBAS software (Wu, Mao, Cai, Luo, & Wei, 2006; Xie et al., 2011). Genes of the chicken were set as the background.

### 2.7 Protein structure determination

From the PSGs identified with PAML, we take *MB* for example to depict the protein and visualize the amino acid changes. The crystal structure of *MB* was obtained from SWISS‐MODEL (Schwede, Kopp, Guex, & Peitsch, 2003). The cartoon and surface representations of gene mutants were visualized through PDB files, which were converted from PQR format with PDB2PQR server (Unni et al., 2011). Furthermore, the electrostatic surface potential was visualized through APBS plugin in PyMOL (DeLano, 2002).

## 3 RESULTS

### 3.1 Genome sequencing, assembly, and quality assessment

We sequenced 37.54 Gb paired‐end reads and 54.99 Gb mate‐paired reads derived from a muscle sample of a single wild male blood pheasant and assembled them into 3,898 scaffolds (N50 = 10.88 Mb, total size = 1.04 Gb) with the longest one 47.18 Mb. In addition, BUSCO evaluated that 4,622 orthologous single‐copy genes assembled 94% of intact single‐copy genes demonstrating high assembly integrity and sequencing uniformity (Table S2).

### 3.2 Genome characterization

The GC content of the blood pheasant genome was approximately 41.23%, similar to other bird species such as red junglefowl and zebra finch. Using homologous sequence alignment and de novo prediction, we identified 109.92 Mb (10.62%) of the repeats in the genome, including DNA elements (1.15%) and retrotransposon (8.09%). Common retrotransposon classes were long interspersed elements (LINEs) (6.85%), long terminal repeat (LTR) (1.19%), and short terminal repeat (SINE) (0.05%) (Table 1). Gene prediction resulted in a total of 17,209 protein‐coding genes (PCGs) for blood pheasant. We found that 16,003 (92.99%) out of 17,209 identified PCGs were well supported by public protein databases (TrEMBL, SwissProt, GO, and KEGG) for the blood pheasant (Table S3). Totally, we identified 279,037 perfect SSRs in the blood pheasant (Table 2) which was lower than that in the buff‐throated partridge (Zhou et al., 2020).

**Table 1:** Repetitive elements’ statistics in the genome of the blood pheasant

Type of repeats	Subfamily	Number of elements	Length occupied (bp)	Percentage in the genome (%)
SINEs		4,888	545,039	0.05
	ALUs	21	1,518	0.00
	MIRs	2,427	242,510	0.02
LINEs		257,132	70,861,874	6.85
	LINE1	3,652	246,561	0.02
	LINE2	4,135	465,781	0.04
	L3/CR1	244,431	69,766,658	6.74
LTR elements		51,968	12,331,241	1.19
	ERVL	28,992	10,153,380	0.98
	ERVL‐MaLRs	26	1,327	0.00
	ERV_classI	3,460	717,757	0.07
	ERV_classII	1,492	253,649	0.02
DNA elements		59,490	11,865,539	1.15
	hAT‐Charlie	16,027	5,549,270	0.54
	TcMar‐Tigger	304	44,313	0.00
Unclassified		5,410	649,744	0.06

Abbreviations: DNA, DNA transposons; LINE, Long Interspersed Nuclear Elements; LTR, Long Terminal Repeated Elements; SINE, Short Interspersed Nuclear Elements.

**Table 2:** The summary of perfect microsatellites detected in the blood pheasant

Type	Counts	Length (bp)	Percent (%)	Average Length (bp)	Relative Abundance (loci/Mb)	Relative Density (bp/Mb)
Mono‐	193,796	3,116,485	69.45	16.08	188.93	3,038.21
Di‐	22,523	418,868	8.07	18.6	21.96	408.35
Tri‐	18,761	338,517	6.72	18.04	18.29	330.01
Tetra‐	29,322	686,952	10.51	23.43	28.59	669.7
Penta‐	12,120	493,915	4.34	40.75	11.82	481.51
Hexa‐	2,515	113,868	0.9	45.28	2.45	111.01

### 3.3 Phylogeny and divergence of Phasianidae species

**FIGURE 2:**
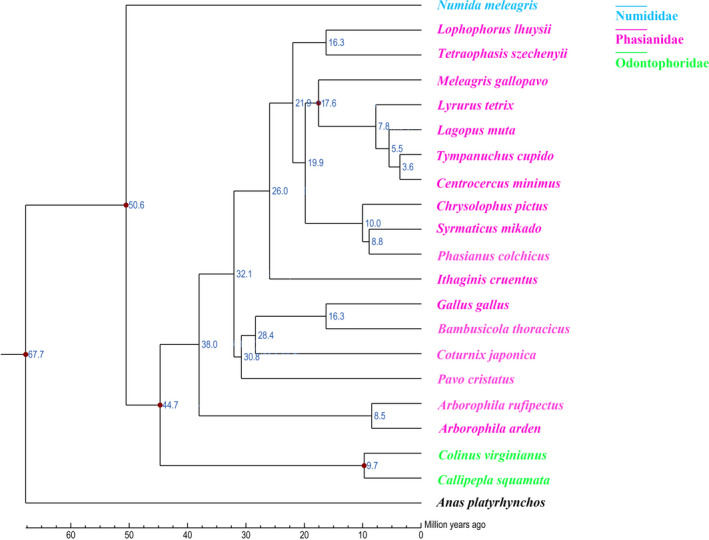
Phylogenetic tree constructed using one‐to‐one orthologous genes. The time lines indicated divergence times among the species

A total of 1,550 one‐to‐one orthologous genes were obtained to reconstruct the phylogenetic tree. The most species‐rich family within Galliformes, Phasianidae, recovered robust relationships among 17 species in the phylogenetic analysis (Figure 2). In general, there were three major clades within Phasianidae. The earliest divergence was between the Hill partridge (*Arborophila* spp.) and the other phasianids. The second one included most of the pheasants, the turkey and the grouse. This group has been designated the “erectile clade” (Kimball & Braun, 2008), and this study extended those findings by including the blood pheasant in the erectile clade. In addition to the strongly supported *Arborophila* and erectile clade, there was a third clade that only received marginal support. This third clade comprised red junglefowl (*Gallus gallus*), Indian peafowl (*Pavo cristatus*), Chinese bamboo partridge (*Bambusicola thoracius*), and Japanese quail (*Coturnix japonica*). Both relationships of members in each group and relationships among the groups receive strong support (Figure 2).

### 3.4 Gene families and positive selection

**FIGURE 3:**
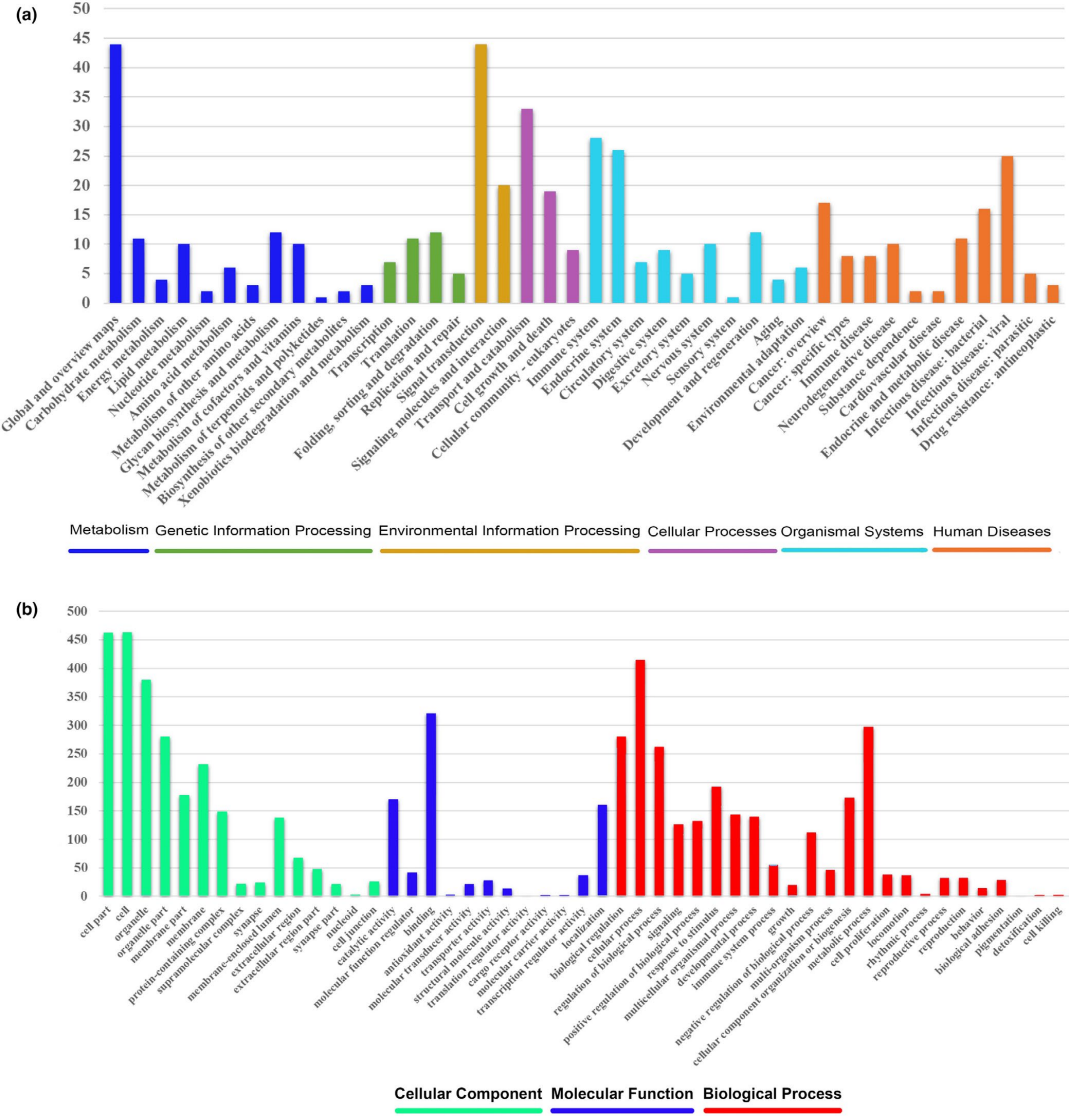
Functional distribution of positively selected genes (PSGs). (a) Functional distribution of PSGs according to the KEGG pathway database. The *x*‐axis illustrated the KEGG functional categories, while the number of genes in each category was plotted on the *y*‐axis. (b) Functional distribution of PSGs according to the gene ontology (GO) database. The x‐axis revealed the GO functional categories, while the number of genes in each category was plotted on the y‐axis

Based on nine avian genomes, a total of 14,009 gene families were detected, of which 5,444 represented 1:1 orthologous gene families. We found that 550 of the 5,444 orthologous genes were under positive selection in blood pheasant. We identified biochemical pathways represented by the PSGs. The KEGG annotation of the PSGs suggested that they were involved in 51 pathways related to metabolism (53 genes), genetic information processing (32 genes), environmental information processing (54 genes), cellular processes (57 genes), organismal systems (70 genes), and human diseases (54 genes) (Figure 3a). The Gene Ontology (GO) annotation classified the PSGs into 3 categories: molecular functions, cellular components, and biological processes (Figure 3b). Molecular functions included genes mainly involved in binding (321 genes; GO:0005488) and catalytic activity (170 genes; GO:0003824). Genes related to cellular components were primarily cell (463 genes; GO:0005623), cell part (462 genes; GO:0044464), and organelle (380 genes; GO:0043226). Biological process genes were mainly involved in cellular process (415 genes; GO:0009987), metabolic process (297 genes; GO:0008152), and biological regulation (280 genes; GO:0065007). The distribution of GO annotations in different functional categories showed a substantial diversity of PSGs. In particular, we found 17 PSGs annotated in response to hypoxia (GO:0001666) and/or response to UV (GO:0009411) have blood pheasant‐specific missense mutations (Table 3).

**Table 3:** Blood pheasant‐specific missense mutations of PSGs associated with high‐altitude adaptation

Gene	Gene description	Position	Amino acid (blood pheasant‐chicken)	Biological category
*MB*	Myoglobin	41	Q‐L	Response to hypoxia
		56	L‐M	
*NARFL*	Cytosolic Fe‐S cluster assembly factor NARFL	343	T‐P	
*PAM*	Peptidyl‐glycine alpha‐amidating monooxygenase	605	E‐K	
		606	K‐E	
		668	L‐S	
*APOLD1*	Apolipoprotein L domain‐containing protein 1	60	M‐L	
*EGR1*	Early growth response protein 1	492	V‐T	
*CD38*	ADP‐ribosyl cyclase/cyclic ADP‐ribose hydrolase 1	81	H‐D	
		270	R‐E	
*APAF1*	Apoptotic protease‐activating factor 1	700	I‐V	
*TNFRSF1A*	Tumor necrosis factor receptor superfamily member 1A	409	E‐Q	
*VCAM1*	Vascular cell adhesion protein 1	84	S‐P	
*CASP3*	Caspase‐3	118	R‐K	Response to hypoxia Response to UV
*RO60*	60 kDa SS‐A/Ro ribonucleoprotein	220	V‐A	Response to UV
*PRIMPOL*	DNA‐directed primase/polymerase protein	24	I‐L	
		56	R‐K	
*DDB2*	DNA damage‐binding protein 2	346	Y‐C	
*ERCC8*	DNA excision repair protein ERCC‐8	315	T‐S	
*ZRANB3*	DNA annealing helicase and endonuclease ZRANB3	351	I‐M	
*ECRR6*	DNA excision repair protein ERCC‐6	1,207	R‐K	

**FIGURE 4:**
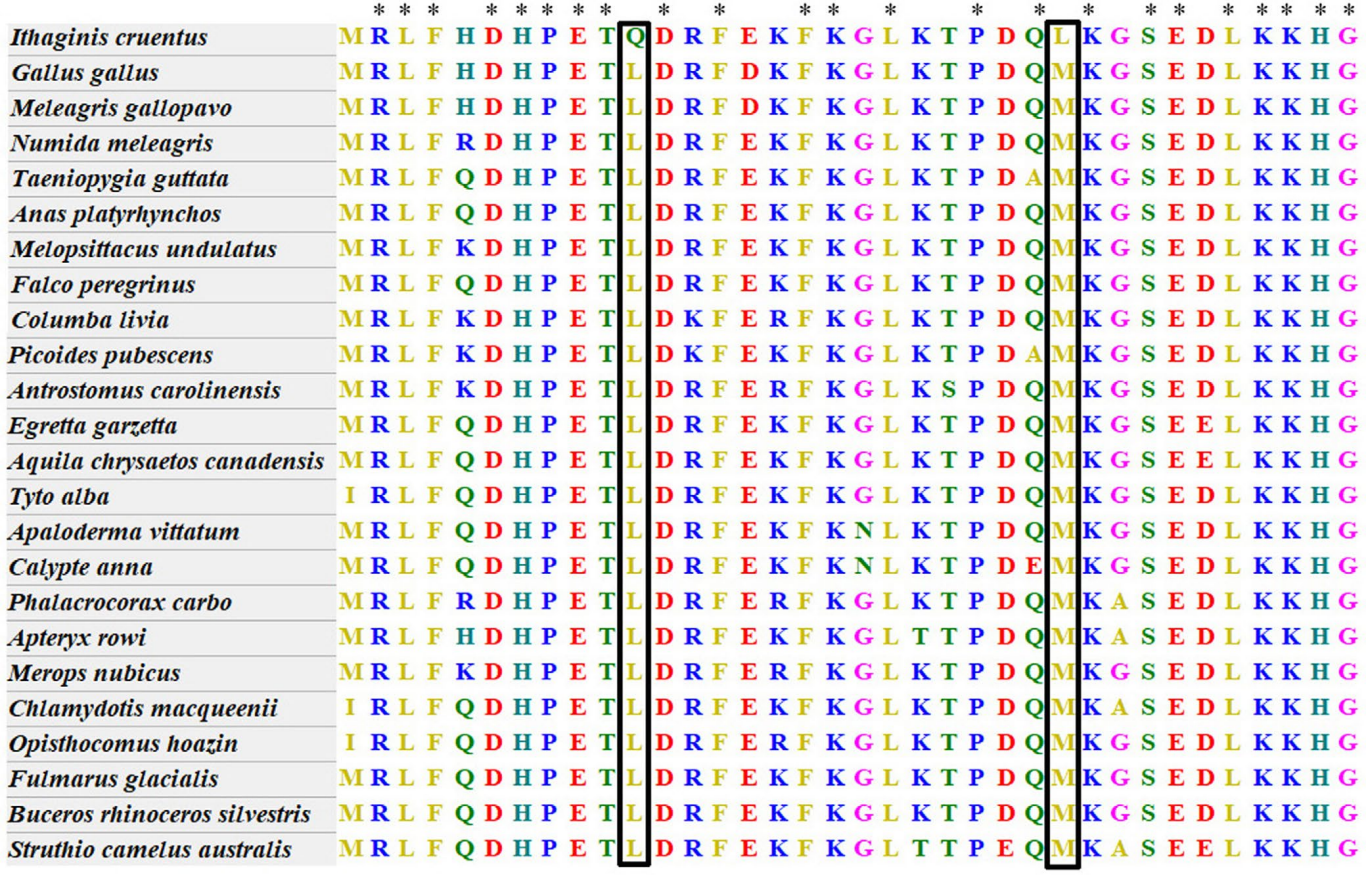
Amino acid sequence alignment of the blood pheasant‐specific missense mutation in Myoglobin (*MB*). The missense mutation in *MB* was marked within rectangle. The asterisk means all species have the same amino acid type at this position

**FIGURE 5:**
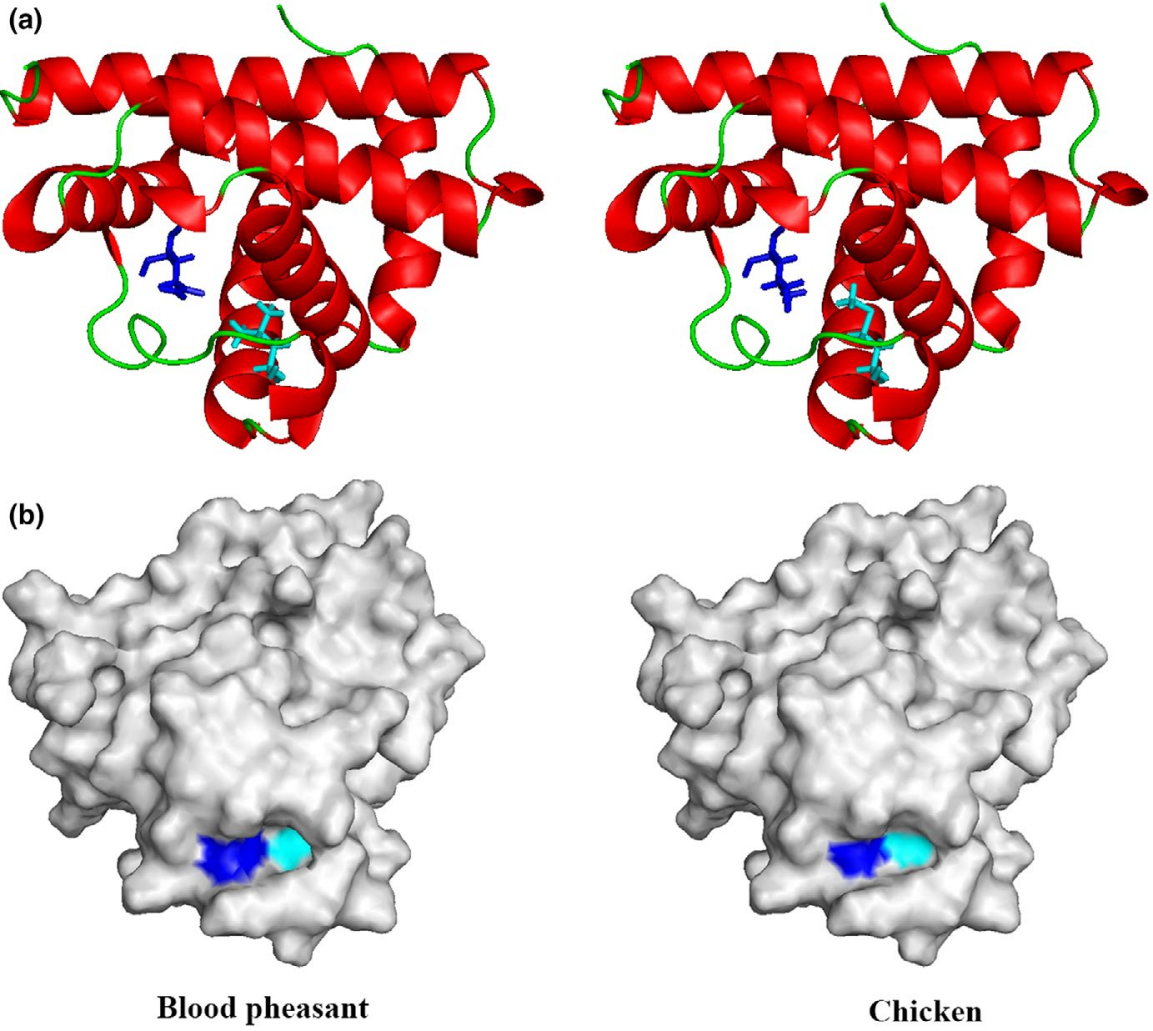
Visualization of non‐mutated and mutated *MB*. (a) Altered amino acids were shown in non‐mutant and mutant *MB* protein models. (b) In the surface of non‐mutant and mutant *MB*, mutation sites were colored as blue and cyan

We further performed GO enrichment with all the PSGs. GO enrichment identified significant overrepresentation of blood pheasant PSGs associated with high‐altitude adaptation (Table S4). A total of 20 PSGs were enriched in mitochondrion (GO:0005739; corrected *p*‐value = .01) which was pivotal to coping with the freezing temperature at high‐altitude environment. Positively selected genes, which could help us understand the genetic basis of specific environmental adaptation, had a significantly higher ratio of nonsynonymous substitutions to synonymous substitutions than other genes in the blood pheasant. After the examination of the PSGs identified in the blood pheasant, we found 16 PSGs containing blood pheasant‐specific missense mutations which validated via comparison to other accessible amino acid sequences of other birds, of which, particularly important, *MB* (Myoglobin) was directly correlative with high‐altitude adaptation and had two blood pheasant missense mutations (Leu41Gln and Met56Leu) (Figure 4 and Table 3). We assessed damaging effects of Leu41Gln and Met56Leu in *MB* on the protein structure, which revealed the changes in cartoon presentation and surface presentation (Figure 5).

## 4 DISCUSSION

### 4.1 Genome feature comparison of Phasianidae species

The NCBI included 8 Phasianidae species genomes—*Syrmaticus mikado*, *Pavo cristatus*, *Chrysolophus pictus*, *Phasianus colchicus*, *Bambusicola thoracicus*, *Coturnix japonica*, *Meleagris gallopavo,* and *Gallus Gallus*. The genome sizes of 8 species ranged from 0.90 to 1.20 Gb, of which the largest was *Meleagris gallopavo* (1.12 Gb) (Dalloul et al., 2010) and the smallest was *Coturnix japonica* (0.93 Gb) (Nishibori, Hayashi, Tsudzuki, Yamamoto, & Yasue, 2001). The genome size of the blood pheasant was 1.04 Gb. Indeed, the genome size of the Phasianidae and even the whole birds has not changed much, all around 1 Gb. *Gallus Gallus* was the best assembly Phasianidae genome with Scaffold N50 reached to 90.11 Mb, whereas the *Bambusicola thoracicus* is only 0.013 Mb (Shen, Shi, Sun, & Zhang, 2009). The Scaffold N50 of the blood pheasant genome we assembled was 10.88 Mb, only smaller than that of *Gallus Gallus* and *Syrmaticus mikado* (Scaffold N50 = 11.32 Mb) (Lee et al., 2018). In addition, the GC rate of most Phasianidae species is 41% including the blood pheasant.

### 4.2 Phylogenetic relationships within Phasianidae

Mitochondrial genes and nuclear genes have been widely used in animal phylogenetic studies, but many studies have shown that the topologies of phylogenetic trees constructed by them may be different (Shaw, 2002; Sota & Vogler, 2001). Mitochondrial genes belong to single copy, strict maternal inheritance, and lack of recombination, so it is very convenient to apply in evolutionary analysis. However, different mitochondrial genes have different evolutionary rates and are subject to different selection pressures, so single gene phylogenetic trees may produce different results. Nuclear genes are relatively conservative and slow in evolution, so they are more suitable for solving phylogenetic problems of higher taxa. But nuclear genes have exchangeable nuclear recombination, as in Phasianidae, where interspecific hybridization is common, so nuclear genes can lead to false conclusions in evolutionary analysis. In addition, most nuclear genes have alleles, which alleles to choose in sequence analysis is also a dilemma. In this study, we obtained the largest phylogenetic tree of Phasianidae species on the basis of whole genomes to date.

The classification of turkey has long been controversial, and the traditional classification classifies turkeys into the Meleagrididae *Meleagris*. Based on the analysis of 102 morphologic traits, turkey and Phasianidae were grouped into monophyletes (Dyke, Gulas, & Crowe, 2003). Part of the mitochondrial genetic phylogenetic tree supports turkey and partridge as sister groups (Crowe et al., 2006). Analysis of the four nuclear genes and two mitochondrial genes together showed that turkey and Koklass pheasant (*Pucrasia macrolopha*) were more closely related than other species (Kimball & Braun, 2008). In this study, it was found that turkey should be classified into Phasianidae and closely related to the grouse. Some of the recent researches were consistent with our conclusion, even placing turkeys and grouses sister to each other (Shen et al., 2010; Wang et al., 2013).

The phylogenetic tree in this study did not support the previous division of *Tetraophasis* spp. into the related genera of *Arborophila* spp. or *Tetraonidae* (Potapov, 2002). *Tetraophasis* spp. had not sex dimorphism, a typical characteristic of partridges. The reason why it was similar to grouse in attribute and habit was that they all lived in the extreme climate of high mountains. The morphological and behavioral characteristics might eventually lead to convergence through adaptive evolution. Our results supported that *Lophophorus lhuysii* and *Tetraophasis szechenyii* belonged to sister branches. Previous studies have shown that their differentiation time was same (2.8–3.0 Mya) (Meng, Dai, Ran, Li, & Yue, 2008). Furthermore, they are mainly distributed in the Himalayas and Hengduan Mountains where the geographical distribution overlaps. From the above, molecular biology, divergence time, and geographical distribution reveal that they are sister genera. Combined with the previous results, *Gallus gallus* and *Bambusicola thoracius* in the same branch, all showed that pheasants and partridges were not monophyletic.

The phylogenetic status of blood pheasant was variable. Dyck used morphogenesis to classify Blood Pheasant into perdicini, for its rectrices molted from the center to the outside, the same as the perdicini (Dyck, 1992). Johnsgard (1999) attributed the blood pheasant to phasianini. Both the protein sequence and the nucleic acid sequence of Cyt b gene showed that the phasianini and the blood pheasant clustered together (Kimball, Braun, Zwartjies, Crowe, & Ligon, 1999). In our evolutionary tree, blood pheasant was a separate branch, which was inconsistent with the previous classification of blood pheasant as pheasants or partridges. Therefore, although the whole phasiandae was monophyletic, neither the pheasants nor the partridges were monophyletic, and they were intermingled in our phylogenetic tree.

### 4.3 Genetic mechanisms of high‐altitude adaptation

Testing for positive selection is often difficult because positive selection tends to operate at several sites and in a short evolutionary time, and signals may be overwhelmed by ubiquitous negative selection. In this paper, we used the branch‐site model in CODEML program from PAML package (Yang, 2007) to detect whether the gene received positive selection in the blood pheasant branch. In this test, the branches of the tree are priori divided into foreground and background lineages, and a likelihood ratio test (LRT) is constructed by comparing a model that allows positive selection of foreground lineages with a model that does not allow positive selection. Its main advantage is the implementation of a rich evolutionary model that can be used to estimate the parameters in a sequential evolutionary model and to test interesting biological hypotheses. Its program, CODEML, can estimate the ratio of synonyms and nonsynonyms (d (N) and d (S)) between two protein‐coding DNA sequences and infer Darwinian selection from phylogenetic comparisons of protein‐coding genes. However, the computer simulation shows that the test is sensitive to the underlying model, and when the model assumptions are violated, the null assumptions of no selection may be rejected frequently, resulting in false positives (Zhang, 2004). If some sites evolve under the negative selection of background lineages, but experience a relaxation of constraints on foreground lineages, the test may be misled into wrongly rejecting the null neutral model (Zhang et al., 2005).

We found that ten PSGs (*MB*, *NARFL*, *PAM*, *APOLD1*, *EGR1*, *CD38*, *APAF1*, *TNFRSF1A*, *VCAM1*, and *CASP3*) with blood pheasant‐specific missense mutations were associated with response to hypoxia. *MB* is a multifunctional heme protein that specializes in oxygen transport, storage, and buffering (Galluzzo, Pennacchietti, Rosano, Comoglio, & Michieli, 2009). The interaction between myoglobin and mitochondria was one of the important mechanisms of hypoxia adaptation: Oxygenated myoglobin was rapidly deoxidized during muscle hypoxia, which increased the oxygen pressure of capillaries and formed a concentration gradient, while its own saturation decreased, closed to the cell membrane and promoted oxygen diffusion. Meanwhile, the density of mitochondria and the cross‐sectional area of muscle fiber decreased correspondingly, reducing the oxygen demand and the distance of oxygen diffusion within muscle fibers, and promoting the adaptation of animals to hypoxia (Jaspers et al., 2014). Previous study found that hypoxia markedly increased the expression of *EGR1* (Nishi, Nishi, & Johnson, 2002). Hypoxia was reported to cause increased *APAF1* protein expression (Chen et al., 2014). Under hypoxia, cells underwent a number of column biochemical changes to accommodate this condition. Some protective stress proteins’ expression were enhanced, and *NARFL* could be directly used as an indicator of oxidative stress, which was due to the fact that *NARF* has been confirmed to be a major antioxidant stress component. In the case of increased levels of oxidative stress, *NARFL* expression increased to resist oxygen free radicals, whereas in the high oxygen condition after the knockdown of *NARFL*, the cell line showed increased death and increased sister chromosome breaks (Corbin, Rockx, Oostra, Joenje, & Dorsman, 2015). Therefore, *NARFL* mutation might cause a decrease in antioxidant stress levels, causing lung damage and malformation of the lung vessels (Kim & Byzova, 2014). In addition, HIF‐1 (hypoxia‐inducing factor 1) acted as a master regulator of numerous hypoxia‐inducible genes under hypoxic conditions. Some studies have also confirmed that *NARFL* was an activity regulator of *HIF‐1*, whereas the knockdown of *NARFL* was demonstrated to be able to upregulate the protein levels of *HIF* and increase their promoter activity and transcription in normoxic and hypoxic states (Huang et al., 2007). CD38 was the target gene of *HIF‐1* and promoted smooth muscle cell (SMCs) proliferation by inhibiting *SIRT1* expression (Li et al., 2011). It could also increase oxygen delivery by mediating Ca^2+^ concentration to regulate the contraction of tracheal smooth muscle (Kuemmerle, Murthy, & Makhlouf, 1998).

Furthermore, a total of seven PSGs (*RO60*, *PRIMPOL*, *DDB2*, *ERCC8*, *ZRANB3*, *ECRR6*, and *CASP3*) which were related to response to UV were found to have blood pheasant‐specific missense mutations. Location of *RO60* was affected by UV irradiation and oxidative stress (Chen et al., 2003). *PRIMPOL* was identified as a factor that is regulated by Rad51 to prevent dysregulated elongation after UV irradiation (Vallerga, Mansilla, Federico, Bertolin, & Gottifredi, 2015). UV light generated T‐T pyrimidine‐pyrimidone photoproducts (6‐4 PPs) in DNA, which were highly distorting and could not be efficiently bypassed by any individual mammalian DNA polymerase in vitro. *PRIMPOL* could be incorporated opposite and extended from a templated 6‐4 PP, possibly mediated by its ability to catalyze translesion synthesis (TLS) of these lesions. *PRIMPOL* was a competent TLS polymerase, bypassing specific oxidative and UV‐induced DNA lesions (Torregrosa‐Muñumer et al., 2017). Previous study indicated that *DDB2* was a modulator of UV‐induced apoptosis and that UV resistance could be overcome by inhibition of *DDB2* (Sun, Kamarajan, Huang, & Chao, 2002). *DDB2* was able to identify CPD (cyclobutane pyrimidine dimers) induced by UV, forming CRL4‐DDB2 subunits with the E3 ubiquitin ligase complex to initiate the entire repair pathway and remove lesions through ubiquitination (Han et al., 2017). Low expression of the *DDB2* gene in cells would lead to the loss of *DDB2* protein binding and scavenge UV‐mediated damage (Bommi, Ravindran, Raychaudhuri, & Bagchi, 2018). *ERCC8* and *ERCC6* encoded proteins involved in the transcription‐coupled DNA repair pathway (Laugel et al., 2010). The most prominent feature of *ERCC8* was that it has multiple WD 40 repeating structures, which were the scaffold for protein–protein interaction and necessary for the construction of β helix structure (Koch et al., 2014). *ERCC6* participated in nuclear and mitochondrial DNA oxidative damage repair through recruiting NER (nucleotide excision repair) factor from DNA damage site (Dianov, Bischoff, Sunesen, & Bohr, 1999).

## 5 CONCLUSIONS

Our results provided the largest phylogenetic tree of Phasianidae species based on complete genomes. We found 10 hypoxia‐related PSGs in the blood pheasant. These genes potentially enhanced function of the blood pheasant under hypoxic conditions by diverse pathways. Besides the hypoxia adaptation, seven PSGs required for high‐altitude survival related to response to UV radiation were determined in blood pheasant, which was likely as a result of the increased exposure of blood pheasant to UV radiation. There is no doubt that the blood pheasant can be selected as an example for studying the process of adaptation to high‐altitude environments.

## CONFLICT OF INTEREST

The authors declare that they have no competing interests.

## AUTHOR CONTRIBUTIONS

**Chuang Zhou:** Conceptualization (equal); Formal analysis (equal); Project administration (equal); Supervision (equal); Writing‐original draft (equal); Writing‐review & editing (equal). **Yi Liu:** Formal analysis (equal); Writing‐original draft (equal); Writing‐review & editing (equal). **Lu Qiao:** Formal analysis (equal); Writing‐review & editing (equal). **Yang Liu:** Writing‐original draft (equal); Writing‐review & editing (equal). **Nan Yang:** Conceptualization (equal); Project administration (equal); Supervision (equal); Writing‐review & editing (equal). **Yang Meng:** Conceptualization (equal); Project administration (equal); Supervision (equal); Writing‐review & editing (equal). **Bisong Yue:** Conceptualization (equal); Project administration (equal); Supervision (equal); Writing‐review & editing (equal).

## Supporting information

Table S1

Table S2

Table S3

Table S4

## Data Availability

Genome and DNA sequencing data of the blood pheasant are available at NCBI BioProject under accession number PRJNA595564. The datasets analyzed during the current study are available and can be found in Dryad Digital Repository: https://doi.org/10.5061/dryad.g79cnp5mb.

## References

[ece36782-bib-0001] Ai, H. , Yang, B. , Li, J. , Xie, X. , Chen, H. , & Ren, J. (2014). Population history and genomic signatures for high‐altitude adaptation in Tibetan pigs. BMC Genomics, 15(1), 834 10.1186/1471-2164-15-834 25270331 PMC4197311

[ece36782-bib-0002] Altschul, S. F. , Madden, T. L. , Schäffer, A. A. , Zhang, J. , Zhang, Z. , Miller, W. , & Lipman, D. J. (1997). Gapped BLAST and PSI‐BLAST: A new generation of protein database search programs. Nucleic Acids Research, 25, 3389–3402. 10.1093/nar/25.17.3389 9254694 PMC146917

[ece36782-bib-0003] Armstrong, M. H. , Braun, E. L. , & Kimball, R. T. (2001). Phylogenetic utility of avian ovomucoid intron G: A comparison of nuclear and mitochondrial phylogenies in Galliformes. The Auk, 118(3), 799–804. 10.2307/4089949

[ece36782-bib-0004] Ashburner, M. , Ball, C. A. , Blake, J. A. , Botstein, D. , Butler, H. , Cherry, J. M. , … Harris, M. A. (2000). Gene ontology: Tool for the unifcation of biology. Nature Genetics, 25, 25. 10802651 10.1038/75556 PMC3037419

[ece36782-bib-0005] Birney, E. , Clamp, M. , & Durbin, R. (2004). GeneWise and genomewise. Genome Research, 14, 988–995. 10.1101/gr.1865504 15123596 PMC479130

[ece36782-bib-0006] Boeckmann, B. , Bairoch, A. , Apweiler, R. , Blatter, M. C. , Estreicher, A. , Gasteiger, E. , … Pilbout, S. (2003). The SWISS‐PROT protein knowledgebase and its supplement TrEMBL in (2003). Nucleic Acids Research, 31, 365–370. 10.1093/nar/gkg095 12520024 PMC165542

[ece36782-bib-0007] Boetzer, M. , Henkel, C. V. , Jansen, H. J. , Butler, D. , & Pirovano, W. (2010). Scaffolding pre‐assembled contigs using SSPACE. Bioinformatics, 27, 578–579. 10.1093/bioinformatics/btq683 21149342

[ece36782-bib-0008] Bommi, P. V. , Ravindran, S. , Raychaudhuri, P. , & Bagchi, S. (2018). DDB2 regulates epithelial‐to‐mesenchymal transition (EMT) in oral/head and neck squamous cell carcinoma. Oncotarget, 9(78), 34708 10.18632/oncotarget.26168 30410671 PMC6205178

[ece36782-bib-0009] Burge, C. , & Karlin, S. (1997). Prediction of complete gene structures in human genomic DNA. Journal of Molecular Biology, 268(1), 78–94. 10.1006/jmbi.1997.0951 9149143

[ece36782-bib-0010] Chen, Q. , Xu, J. , Li, L. , Li, H. , Mao, S. , Zhang, F. , … Zhang, Q. (2014). MicroRNA‐23a/b and microRNA‐27a/b suppress Apaf‐1 protein and alleviate hypoxia‐induced neuronal apoptosis. Cell Death & Disease, 5(3), e1132 10.1038/cddis.2014.92 24651435 PMC3973202

[ece36782-bib-0011] Chen, X. , Smith, J. D. , Shi, H. , Yang, D. D. , Flavell, R. A. , & Wolin, S. L. (2003). The Ro autoantigen binds misfolded U2 small nuclear RNAs and assists mammalian cell survival after UV irradiation. Current Biology, 13(24), 2206–2211. 10.1016/j.cub.2003.11.028 14680639

[ece36782-bib-0012] Cheng, T. H. (1978). Fauna Sinica, Aves, Vol. 4: Galliformes. Beijing, China: Science Press (in Chinese).

[ece36782-bib-0013] Corbin, M. V. , Rockx, D. A. , Oostra, A. B. , Joenje, H. , & Dorsman, J. C. (2015). The iron‐sulfur cluster assembly network component NARFL is a key element in the cellular defense against oxidative stress. Free Radical Biology and Medicine, 89, 863–872. 10.1016/j.freeradbiomed.2015.08.026 26456054

[ece36782-bib-0014] Crowe, T. M. , Bowie, R. C. K. , Bloomer, P. , Mandiwana, T. G. , Hedderson, T. A. J. , Randi, E. , … Wakeling, J. (2006). Phylogenetics, biogeography and classification of, and character evolution in, gamebirds (Aves: Galliformes): Effects of character exclusion, data partitioning and missing data. Cladistics, 22(6), 495–532. 10.1111/j.1096-0031.2006.00120.x 34892896

[ece36782-bib-0015] Cui, K. , Li, W. , James, J. G. , Peng, C. , Jin, J. , Yan, C. , … Yue, B. (2019). The first draft genome of *Lophophorus*: A step forward for Phasianidae genomic diversity and conservation. Genomics, 111(6), 1209–1215. 10.1016/j.ygeno.2018.07.016 30063977

[ece36782-bib-0016] Dalloul, R. A. , Long, J. A. , Zimin, A. V. , Aslam, L. , Beal, K. , Ann Blomberg, L. , … Crooijmans, R. P. M. A. (2010). Multi‐platform next‐generation sequencing of the domestic Turkey (Meleagris gallopavo): Genome assembly and analysis. PLoS Biology, 8(9), e1000475 10.1371/journal.pbio.100475 20838655 PMC2935454

[ece36782-bib-0017] DeLano, W. L. (2002). Pymol: An open‐source molecular graphics tool. CCP4 Newsletter on Protein Crystallography, 40, 82–92.

[ece36782-bib-0018] Dianov, G. , Bischoff, C. , Sunesen, M. , & Bohr, V. A. (1999). Repair of 8‐oxoguanine in DNA is deficient in Cockayne syndrome group B cells. Nucleic Acids Research, 27(5), 1365–1368. 10.1093/nar/27.5.1365 9973627 PMC148325

[ece36782-bib-0019] Doyle, J. M. , Katzner, T. E. , Bloom, P. H. , Ji, Y. , Wijayawardena, B. K. , & DeWoody, J. A. (2014). The genome sequence of a widespread apex predator, the golden eagle (*Aquila chrysaetos*). PLoS One, 9(4). 10.1371/journal.pone.0095599 PMC3997482 24759626

[ece36782-bib-0020] Du, L. , Zhang, C. , Liu, Q. , Zhang, X. , & Yue, B. (2018). Krait: An ultrafast tool for genome‐wide survey of microsatellites and primer design. Bioinformatics, 34(4), 681–683. 10.1093/bioinformatics/btx665 29048524

[ece36782-bib-0021] Dyck, J. (1992). Reflectance spectra of plumage areas colored by green feather pigments. The Auk, 109, 293–301. 10.2307/4088197

[ece36782-bib-0022] Dyke, G. J. , Gulas, B. E. , & Crowe, T. M. (2003). Suprageneric relationships of galliform birds (Aves, Galliformes): A cladistic analysis of morphological characters. Zoological Journal of the Linnean Society, 137(2), 227–244. 10.1046/j.1096-3642.2003.00048.x

[ece36782-bib-0023] Galluzzo, M. , Pennacchietti, S. , Rosano, S. , Comoglio, P. M. , & Michieli, P. (2009). Prevention of hypoxia by myoglobin expression in human tumor cells promotes differentiation and inhibits metastasis. The Journal of Clinical Investigation., 119(4), 865–875. 10.1172/JCI36579 19307731 PMC2662552

[ece36782-bib-0024] Ge, R.‐L. , Cai, Q. , Shen, Y.‐Y. , San, A. , Ma, L. , Zhang, Y. , … Wang, J. (2013). Draft genome sequence of the Tibetan antelope. Nature Communications, 4, 1858 10.1038/ncomms2860 PMC3674232 23673643

[ece36782-bib-0025] Gorkhali, N. A. , Dong, K. , Yang, M. , Song, S. , Kader, A. , Shrestha, B. S. , … Ma, Y. (2016). Genomic analysis identified a potential novel molecular mechanism for high‐altitude adaptation in sheep at the Himalayas. Scientific Reports, 6, 29963 10.1038/srep29963 27444145 PMC4995607

[ece36782-bib-0026] Haas, B. J. , Salzberg, S. L. , Zhu, W. , Pertea, M. , Allen, J. E. , Orvis, J. , … Wortman, J. R. (2008). Automated eukaryotic gene structure annotation using EVidenceModeler and the program to assemble spliced alignments. Genome Biology, 9, 1 10.1186/gb-2008-9-1-r7 PMC2395244 18190707

[ece36782-bib-0027] Han, C. , Zhao, R. , Kroger, J. , He, J. , Wani, G. , Wang, Q. E. , & Wani, A. A. (2017). UV radiation‐induced SUMOylation of DDB2 regulates nucleotide excision repair. Carcinogenesis, 38(10), 976–985. 10.1093/carcin/bgx076 28981631 PMC7446238

[ece36782-bib-0028] Huang, J. , Song, D. , Flores, A. , Zhao, Q. , Mooney, S. M. , Shaw, L. M. , & Lee, F. S. (2007). IOP1, a novel hydrogenase‐like protein that modulates hypoxia‐inducible factor‐1α activity. Biochemical Journal, 401(1), 341–352. 10.1042/bj20060635 16956324 PMC1698691

[ece36782-bib-0029] IUCN . 2012 The IUCN Red List of Threatened Species[EB/OL]. (2012.02)[2012‐10‐17]. http://www.iucnredlist.org

[ece36782-bib-0030] Jaspers, R. T. , Testerink, J. , Della Gaspera, B. , Chanoine, C. , Bagowski, C. P. , & van der Laarse, W. J. (2014). Increased oxidative metabolism and myoglobin expression in zebrafish muscle during chronic hypoxia. Biology Open, 3(8), 718–727. 10.1242/bio.20149167 25063194 PMC4133725

[ece36782-bib-0031] Johnsgard, P. A. (1999). The pheasants of the world, (2nd ed.). New York: Oxford University Press.

[ece36782-bib-0032] Kim, Y. W. , & Byzova, T. V. (2014). Oxidative stress in angiogenesis and vascular disease. Blood, the Journal of the American Society of Hematology, 123(5), 625–631. 10.1182/blood-2013-09-512749 PMC3907751 24300855

[ece36782-bib-0033] Kimball, R. T. , & Braun, E. L. (2008). A multigene phylogeny of Galliformes supports a single origin of erectile ability in non‐feathered facial traits. Journal of Avian Biology, 39(4), 438–445. 10.1111/j.0908-8857.2008.04270.x

[ece36782-bib-0034] Kimball, R. C. , Braun, E. L. , Zwartjies, P. W. , Crowe, T. M. , & Ligon, J. D. (1999). A molecular phylogeny of the pheasants and partridges suggests that these lineages are not monophyletic. Molecular Phylogenetics and Evolution, 11, 38–54. 10.1006/mpev.1998.0562 10082609

[ece36782-bib-0035] Koch, S. , Garcia Gonzalez, O. , Assfalg, R. , Schelling, A. , Schäfer, P. , Scharffetter‐Kochanek, K. , & Iben, S. (2014). Cockayne syndrome protein A is a transcription factor of RNA polymerase I and stimulates ribosomal biogenesis and growth. Cell Cycle, 13(13), 2029–2037. 10.4161/cc.29018 24781187 PMC4111694

[ece36782-bib-0036] Kuemmerle, J. F. , Murthy, K. S. , & Makhlouf, G. M. (1998). Longitudinal smooth muscle of the mammalian intestine. Cell Biochemistry and Biophysics, 28(1), 31–44. 10.1007/bf02738308 9386891

[ece36782-bib-0037] Laugel, V. , Dalloz, C. , Durand, M. , Sauvanaud, F. , Kristensen, U. , Vincent, M. C. , … Tobias, E. S. (2010). Mutation update for the CSB/ERCC6 and CSA/ERCC8 genes involved in Cockayne syndrome. Human Mutation, 31(2), 113–126. 10.1002/humu.21154 19894250

[ece36782-bib-0038] Lee, C.‐Y. , Hsieh, P.‐H. , Chiang, L.‐M. , Chattopadhyay, A. , Li, K.‐Y. , Lee, Y.‐F. , … Chuang, E. Y. (2018). Whole‐genome de novo sequencing reveals unique genes that contributed to the adaptive evolution of the Mikado pheasant. GigaScience, 7(5), giy044, –. 10.1093/gigascience/giy04 PMC5941149 29722814

[ece36782-bib-0039] Li, L. , Stoeckert, C. J. , & Roos, D. S. (2003). OrthoMCL: Identification of ortholog groups for eukaryotic genomes. Genome Research, 13, 2178–2189. 10.1101/gr.1224503 12952885 PMC403725

[ece36782-bib-0040] Li, L. I. , Zhang, H.‐N. , Chen, H.‐Z. , Gao, P. , Zhu, L.‐H. , Li, H.‐L. , … Liang, C.‐C. (2011). SIRT1 acts as a modulator of neointima formation following vascular injury in mice. Circulation Research, 108(10), 1180–1189. 10.1161/circresaha.110.237875 21474819

[ece36782-bib-0041] Lohse, M. , Bolger, A. M. , Nagel, A. , Fernie, A. R. , Lunn, J. E. , Stitt, M. , & Usadel, B. (2012). R obi NA: A user‐friendly, integrated software solution for RNA‐Seq‐based transcriptomics. Nucleic Acids Research, 40(W1), W622–W627. 10.1093/nar/gks540 22684630 PMC3394330

[ece36782-bib-0042] Löytynoja, A. , & Goldman, N. (2010). webPRANK: A phylogeny‐aware multiple sequence aligner with interactive alignment browser. BMC Bioinformatics, 11(1), 579 10.1186/1471-2105-11-579 21110866 PMC3009689

[ece36782-bib-0043] Luo, R. , Liu, B. , Xie, Y. , Li, Z. , Huang, W. , Yuan, J. , … Wang, J. (2012). SOAPdenovo2: An empirically improved memory‐efficient short‐read de novo assembler. Gigascience, 1, 18 10.1186/2047-217x-1-18 23587118 PMC3626529

[ece36782-bib-0044] MacKinnon, J. , Phillipps, K. , & He, F. Q. (2000). A field guide to the birds of China, Changsha, China: Hunan Education Press.

[ece36782-bib-0045] Meng, Y. , Dai, B. , Ran, J. H. , Li, J. , & Yue, B. S. (2008). Phylogenetic position of the genus *Tetraophasis* (Aves, Galliformes, Phasianidae) as inferred from mitochondrial and nuclear sequences. Biochemical Systematics and Ecology, 36, 626–637. 10.1016/j.bse.2008.01.007

[ece36782-bib-0046] Moriya, Y. , Itoh, M. , Okuda, S. , Yoshizawa, A. C. , & Kanehisa, M. (2007). KAAS: An automatic genome annotation and pathway reconstruction server. Nucleic Acids Research, 35, W182–W185. 10.1093/nar/gkm321 17526522 PMC1933193

[ece36782-bib-0047] Nishi, H. , Nishi, K. H. , & Johnson, A. C. (2002). Early growth response‐1 gene mediates up‐regulation of epidermal growth factor receptor expression during hypoxia. Cancer Research, 62(3), 827–834. 11830539

[ece36782-bib-0048] Nishibori, M. , Hayashi, T. , Tsudzuki, M. , Yamamoto, Y. , & Yasue, H. (2001). Complete sequence of the Japanese quail (Coturnix japonica) mitochondrial genome and its genetic relationship with related species. Animal Genetics, 32(6), 380–385. 10.1046/j.1365-2052.2001.00795.x 11736810

[ece36782-bib-0049] Pereira, S. L. , & Baker, A. J. (2006). A molecular timescale for galliform birds accounting for uncertainty in time estimates and heterogeneity of rates of DNA substitutions across lineages and sites. Molecular Phylogenetics and Evolution, 38(2), 499–509. 10.1016/j.ympev.2005.07.007 16112881

[ece36782-bib-0050] Potapov, R. L. (2002). Distribution, biology and phylogeny of genus *Tetraophasis* (Elliot, 1872). Russian Journal of Ornithology, 11, 1051–1066.

[ece36782-bib-0051] Qiu, Q. , Zhang, G. , Ma, T. , Qian, W. , Wang, J. , Ye, Z. , … Liu, J. (2012). The yak genome and adaptation to life at high altitude. Nature Genetics, 44, 946 10.1038/ng.2343 22751099

[ece36782-bib-0052] Schwede, T. , Kopp, J. , Guex, N. , & Peitsch, M. C. (2003). Swiss‐model: An automated protein homology‐modeling server. Nucleic Acids Research, 31, 3381–3385. 10.1093/nar/gkg520 12824332 PMC168927

[ece36782-bib-0053] Seabury, C. M. , Dowd, S. E. , Seabury, P. M. , Raudsepp, T. , Brightsmith, D. J. , Liboriussen, P. , … Tizard, I. R. (2013). A multi‐platform draft de novo genome assembly and comparative analysis for the scarlet macaw (*Ara macao*). PLoS One, 8, e62415 10.1371/journal.pone.0062415 23667475 PMC3648530

[ece36782-bib-0054] Shaw, K. L. (2002). Conflict between nuclear and mitochondrial DNA phylogenies of a recent species radiation: What mtDNA reveals and conceals about modes of speciation in Hawaiian crickets. Proceedings of the National Academy of Sciences of the United States of America, 99(25), 16122–16127. 10.1073/pnas.242585899 12451181 PMC138575

[ece36782-bib-0055] Shen, Y. Y. , Dai, K. , Cao, X. , Murphy, R. W. , Shen, X. J. , & Zhang, Y. P. (2014). The updated phylogenies of the Phasianidae based on combined data of nuclear and mitochondrial DNA. PLoS One, 9(4), e95786 10.1371/journal.pone.0095786 24748132 PMC3991718

[ece36782-bib-0056] Shen, Y. Y. , Liang, L. , Sun, Y. B. , Yue, B. S. , Yang, X. J. , Murphy, R. W. , & Zhang, Y. P. (2010). A mitogenomic perspective on the ancient, rapid radiation in the Galliformes with an emphasis on the Phasianidae. BMC Evolutionary Biology, 10(1), 132 10.1186/1471-2148-10-132 20444289 PMC2880301

[ece36782-bib-0057] Shen, Y.‐Y. , Shi, P. , Sun, Y.‐B. , & Zhang, Y.‐P. (2009). Relaxation of selective constraints on avian mitochondrial DNA following the degeneration of flight ability. Genome Research, 19(10), 1760–1765. 10.1101/gr.093138.109 19617397 PMC2765268

[ece36782-bib-0058] Simão, F. A. , Waterhouse, R. M. , Ioannidis, P. , Kriventseva, E. V. , & Zdobnov, E. M. (2015). BUSCO: Assessing genome assembly and annotation completeness with single‐copy orthologs. Bioinformatics, 31, 3210–3212. 10.1093/bioinformatics/btv351 26059717

[ece36782-bib-0059] Smit, A. F. , Hubley, R. , & Green, P. (2010). RepeatMasker open. Retrieved from http://www.repeatmasker.org. Accessed 28 May 2018.

[ece36782-bib-0060] Sota, T. , & Vogler, A. P. (2001). Incongruence of mitochondrial and nuclear gene trees in the carabid beetles Ohomopterus. Systematic Biology, 50(1), 39–59. 10.1093/sysbio/50.1.39 12116593

[ece36782-bib-0061] Stamatakis, A. (2014). RAxML version 8: A tool for phylogenetic analysis and post‐analysis of large phylogenies. Bioinformatics, 30, 1312–1313. 10.1093/bioinformatics/btu033 24451623 PMC3998144

[ece36782-bib-0062] Stanke, M. , Keller, O. , Gunduz, I. , Hayes, A. , Waack, S. , & Morgenstern, B. (2006). AUGUSTUS: Ab initio prediction of alternative transcripts. Nucleic Acids Research, 34(suppl_2), W435–W439. 10.1093/nar/gkl200 16845043 PMC1538822

[ece36782-bib-0063] Sun, N. K. , Kamarajan, P. , Huang, H. , & Chao, C. C. (2002). Restoration of UV sensitivity in UV‐resistant HeLa cells by antisense‐mediated depletion of damaged DNA‐binding protein 2 (DDB2). FEBS Letters, 512(1–3), 168–172. 10.1016/s0014-5793(02)02250-0 11852074

[ece36782-bib-0064] Torregrosa‐Muñumer, R. , Forslund, J. M. E. , Goffart, S. , Pfeiffer, A. , Stojkovič, G. , Carvalho, G. , … Pohjoismäki, J. L. O. (2017). PrimPol is required for replication reinitiation after mtDNA damage. Proceedings of the National Academy of Sciences of the United States of America, 114(43), 11398–11403. 10.1073/pnas.1705367114 29073063 PMC5664498

[ece36782-bib-0065] Unni, S. , Huang, Y. , Hanson, R. M. , Tobias, M. , Krishnan, S. , Li, W. W. , … Baker, N. A. (2011). Web servers and services for electrostatics calculations with APBS and PDB2PQR. Journal of Computational Chemistry, 32, 1488–1491. 10.1002/jcc.21720 21425296 PMC3062090

[ece36782-bib-0066] Vallerga, M. B. , Mansilla, S. F. , Federico, M. B. , Bertolin, A. P. , & Gottifredi, V. (2015). Rad51 recombinase prevents Mre11 nuclease‐dependent degradation and excessive PrimPol‐mediated elongation of nascent DNA after UV irradiation. Proceedings of the National Academy of Sciences of the United States of America, 112(48), E6624–E6633. 10.1073/pnas.1508543112 26627254 PMC4672812

[ece36782-bib-0067] Wang, M.‐S. , Li, Y. , Peng, M.‐S. , Zhong, L. I. , Wang, Z.‐J. , Li, Q.‐Y. , … Zhang, Y.‐P. (2015). Genomic analyses reveal potential independent adaptation to high altitude in Tibetan chickens. Molecular Biology and Evolution, 32(7), 1880–1889. 10.1093/molbev/msv071 25788450

[ece36782-bib-0068] Wang, N. , Kimball, R. T. , Braun, E. L. , Liang, B. , & Zhang, Z. (2013). Assessing phylogenetic relationships among Galliformes: A multigene phylogeny with expanded taxon sampling in Phasianidae. PLoS One, 8(5), e64312 10.1371/journal.pone.0064312 23741315 PMC3669371

[ece36782-bib-0069] Wu, J. , Mao, X. , Cai, T. , Luo, J. , & Wei, L. (2006). KOBAS server: A web‐based platform for automated annotation and pathway identification. Nucleic Acids Research, 34, W720–W724. 10.1093/nar/gkl167 16845106 PMC1538915

[ece36782-bib-0070] Xie, C. , Mao, X. , Huang, J. , Ding, Y. , Wu, J. , Dong, S. , … Wei, L. (2011). KOBAS 2.0: A web server for annotation and identification of enriched pathways and diseases. Nucleic Acids Research, 39, W316–W322. 10.1093/nar/gkr483 21715386 PMC3125809

[ece36782-bib-0071] Yang, Z. (2007). PAML 4: Phylogenetic analysis by maximum likelihood. Molecular Biology and Evolution, 24, 1586–1591. 10.1093/molbev/msm088 17483113

[ece36782-bib-0072] Zeng, T. , Tu, F. , Ma, L. , Yan, C. , Yang, N. , Zhang, X. , … Ran, J. (2013). Complete mitochondrial genome of blood pheasant (*Ithaginis cruentus*). Mitochondrial DNA, 24(5), 484–486. 10.3109/19401736.2013.770498 23521063

[ece36782-bib-0073] Zhang, J. (2004). Frequent false detection of positive selection by the likelihood method with branch‐site models. Molecular Biology and Evolution, 21(7), 1332–1339. 10.1093/molbev/msh117 15014150

[ece36782-bib-0075] Zhang, J. , Nielsen, R. , & Yang, Z. (2005). Evaluation of an improved branch‐site likelihood method for detecting positive selection at the molecular level. Molecular Biology and Evolution, 22(12), 2472–2479. 10.1093/molbev/msi237 16107592

[ece36782-bib-0076] Zhou, C. , James, J. G. , Xu, Y. U. , Tu, H. , He, X. , Wen, Q. , … Yue, B. (2020). Genome‐wide analysis sheds light on the high‐altitude adaptation of the buff‐throated partridge (*Tetraophasis szechenyii*). Molecular Genetics and Genomics, 295(1), 31–46. 10.1007/s00438-019-01601-8 31414227

[ece36782-bib-0077] Zhou, C. , Tu, H. , Yu, H. , Zheng, S. , Dai, B. O. , Price, M. , … Meng, Y. (2019). The draft genome of the endangered sichuan partridge (*Arborophila rufipectus*) with evolutionary implications. Genes, 10(9), 677 10.3390/genes10090677 PMC6770966 31491910

[ece36782-bib-0078] Zhou, C. , Zheng, S. , Jiang, X. , Liang, W. , Price, M. , Fan, Z. , … Yue, B. (2018). First complete genome sequence in *Arborophila* and comparative genomics reveals the evolutionary adaptation of Hainan Partridge (*Arborophila ardens*). Avian Research, 9(1), 45 10.1186/s40657-018-0136-3

