# Supplementary material for: The draft genome of the blood pheasant (Ithaginis cruentus): Phylogeny and high‐altitude adaptation
Source: Ecol Evol. 2020 Sep 28;10(20):11440–52. doi: 10.1002/ece3.6782 (PMC7593199; doi:10.1002/ece3.6782)
Supplement: Supplementary file 1 — Table S1 [file ECE3-10-11440-s001.docx]

**Table S1** Information of genomes used in this study

| Species | Accession/Reference |
| --- | --- |
| *Arborophila ardens* | Zhou et al. 2018 |
| *Arborophila rufipectus* | Zhou et al. 2019 |
| *Bambusicola thoracicus* | GCA_002909625.1 |
| *Callipepla squamata* | GCA_002218305.1 |
| *Centrocercus minimus* | GCA_005890655.1 |
| *Chrysolophus pictus* | GCA_003413605.1 |
| *Colinus virginianus* | GCA_008692595.1 |
| *Coturnix japonica* | GCF_001577835.2 |
| *Gallus gallus* | GCF_000002315.6 |
| *Ithaginis cruentus* | This study |
| *Lagopus muta* | GCA_004320205.1 |
| *Lophophorus lhuysii* | Cui et al. 2019 |
| *Lyrurus tetrix* | GCA_000586395.1 |
| *Meleagris gallopavo* | GCF_000146605.3 |
| *Numida meleagris* | GCF_002078875.1 |
| *Pavo cristatus* | GCA_005519975.1 |
| *Phasianus colchicus* | GCF_004143745.1 |
| *Syrmaticus mikado* | GCA_003435085.1 |
| *Tetraophasis szechenyii* | Zhou et al. 2020 |
| *Tympanuchus cupido* | GCA_001870855.1 |
| *Anas platyrhynchos* | GCF_003850225.1 |
