# Supplementary material for: The draft genome of the blood pheasant (Ithaginis cruentus): Phylogeny and high‐altitude adaptation
Source: Ecol Evol. 2020 Sep 28;10(20):11440–52. doi: 10.1002/ece3.6782 (PMC7593199; doi:10.1002/ece3.6782)
Supplement: Supplementary file 2 — Table S2 [file ECE3-10-11440-s002.docx]

**Table S2** Statistics of the genome completeness of the blood pheasant based on BUSCO benchmark

| BUSCO benchmark | Number | Percentage (%) |
| --- | --- | --- |
| Complete Single-Copy BUSCOs | 4622 | 94.0 |
| Complete Duplicated BUSCOs | 51 | 1.0 |
| Fragmented BUSCOs | 146 | 3.0 |
| Missing BUSCOs | 96 | 2.0 |
| Total BUSCO groups searched | 4915 | 100 |
