# Supplementary material for: The draft genome of the blood pheasant (Ithaginis cruentus): Phylogeny and high‐altitude adaptation
Source: Ecol Evol. 2020 Sep 28;10(20):11440–52. doi: 10.1002/ece3.6782 (PMC7593199; doi:10.1002/ece3.6782)
Supplement: Supplementary file 3 — Table S3 [file ECE3-10-11440-s003.docx]

**Table S3** Functional annotation of the blood pheasant genes

|  |  | Number | Percentage (%) |
| --- | --- | --- | --- |
| Total |  | 17041 | 100 |
| Annotated | SwissProt | 15,030 | 88.20 |
|  |  |  |  |
|  | TrEMBL | 15,644 | 91.80 |
|  |  |  |  |
|  | KEGG | 9,890 | 58.04 |
|  |  |  |  |
|  |  |  |  |
|  | GO | 14,485 | 85.00 |
|  |  |  |  |
|  | Nr | 15,769 | 92.54 |
|  |  |  |  |
|  | all | 15,798 | 92.71 |
|  |  |  |  |
| Unannotated |  | 1,243 | 7.29 |
