# Supplementary material for: The draft genome of the blood pheasant (Ithaginis cruentus): Phylogeny and high‐altitude adaptation
Source: Ecol Evol. 2020 Sep 28;10(20):11440–52. doi: 10.1002/ece3.6782 (PMC7593199; doi:10.1002/ece3.6782)
Supplement: Supplementary file 4 — Table S4 [file ECE3-10-11440-s004.docx]

**Table S4** GO enrichment analysis of positively selected genes in the blood pheasant

| Term | ID | No. of genes | P-Value | Corrected P-Value |
| --- | --- | --- | --- | --- |
| intracellular part | GO:0044424 | 125 | 7.44E-13 | 1.51E-09 |
| intracellular | GO:0005622 | 128 | 1.10E-12 | 1.51E-09 |
| intracellular membrane-bounded organelle | GO:0043231 | 102 | 2.32E-12 | 1.63E-09 |
| cellular process | GO:0009987 | 132 | 2.38E-12 | 1.63E-09 |
| cell part | GO:0044464 | 135 | 2.06E-11 | 9.76E-09 |
| intracellular organelle | GO:0043229 | 108 | 2.46E-11 | 9.76E-09 |
| cell | GO:0005623 | 135 | 2.80E-11 | 9.76E-09 |
| membrane-bounded organelle | GO:0043227 | 107 | 2.85E-11 | 9.76E-09 |
| organelle | GO:0043226 | 113 | 1.60E-10 | 4.89E-08 |
| cytoplasm | GO:0005737 | 94 | 1.80E-10 | 4.95E-08 |
| single-organism process | GO:0044699 | 110 | 6.33E-10 | 1.58E-07 |
| cytoplasmic part | GO:0044444 | 69 | 3.69E-09 | 8.43E-07 |
| metabolic process | GO:0008152 | 98 | 7.21E-09 | 1.52E-06 |
| organic substance metabolic process | GO:0071704 | 93 | 1.10E-08 | 2.15E-06 |
| primary metabolic process | GO:0044238 | 88 | 3.30E-08 | 6.03E-06 |
| cellular metabolic process | GO:0044237 | 88 | 4.82E-08 | 8.26E-06 |
| nucleus | GO:0005634 | 63 | 1.18E-07 | 1.90E-05 |
| macromolecule metabolic process | GO:0043170 | 78 | 2.05E-07 | 3.12E-05 |
| binding | GO:0005488 | 98 | 2.69E-07 | 3.88E-05 |
| single-organism cellular process | GO:0044763 | 90 | 3.18E-07 | 4.36E-05 |
| cellular macromolecule metabolic process | GO:0044260 | 72 | 7.30E-07 | 9.53E-05 |
| establishment of localization | GO:0051234 | 45 | 1.48E-06 | 0.000184 |
| localization | GO:0051179 | 52 | 1.57E-06 | 0.000187 |
| transport | GO:0006810 | 44 | 1.64E-06 | 0.000187 |
| intracellular organelle part | GO:0044446 | 64 | 2.53E-06 | 0.000277 |
| membrane | GO:0016020 | 73 | 2.71E-06 | 0.000286 |
| biological regulation | GO:0065007 | 84 | 3.09E-06 | 0.000314 |
| regulation of cellular process | GO:0050794 | 76 | 5.07E-06 | 0.000493 |
| organelle part | GO:0044422 | 64 | 5.23E-06 | 0.000493 |
| regulation of biological process | GO:0050789 | 79 | 5.39E-06 | 0.000493 |
| membrane part | GO:0044425 | 58 | 7.69E-06 | 0.00068 |
| intrinsic component of membrane | GO:0031224 | 50 | 2.48E-05 | 0.002123 |
| negative regulation of biological process | GO:0048519 | 40 | 3.41E-05 | 0.002835 |
| regulation of metabolic process | GO:0019222 | 49 | 3.65E-05 | 0.002942 |
| organelle organization | GO:0006996 | 35 | 3.95E-05 | 0.003026 |
| regulation of cellular metabolic process | GO:0031323 | 47 | 3.97E-05 | 0.003026 |
| single-organism organelle organization | GO:1902589 | 22 | 4.51E-05 | 0.003343 |
| enzyme binding | GO:0019899 | 19 | 4.83E-05 | 0.003484 |
| endomembrane system | GO:0012505 | 33 | 4.99E-05 | 0.003509 |
| protein binding | GO:0005515 | 50 | 5.18E-05 | 0.00355 |
| integral component of membrane | GO:0016021 | 48 | 6.09E-05 | 0.004076 |
| nuclear part | GO:0044428 | 34 | 8.59E-05 | 0.005611 |
| heterocycle metabolic process | GO:0046483 | 46 | 0.000103 | 0.006602 |
| organic cyclic compound metabolic process | GO:1901360 | 47 | 0.000121 | 0.007412 |
| nucleobase-containing compound metabolic process | GO:0006139 | 45 | 0.000122 | 0.007412 |
| regulation of primary metabolic process | GO:0080090 | 45 | 0.000124 | 0.007412 |
| protein metabolic process | GO:0019538 | 46 | 0.000127 | 0.007412 |
| negative regulation of metabolic process | GO:0009892 | 25 | 0.00013 | 0.007448 |
| cellular aromatic compound metabolic process | GO:0006725 | 46 | 0.000133 | 0.00745 |
| nuclear lumen | GO:0031981 | 30 | 0.000139 | 0.00754 |
| single-organism localization | GO:1902578 | 27 | 0.000144 | 0.00754 |
| single-organism metabolic process | GO:0044710 | 36 | 0.000144 | 0.00754 |
| regulation of macromolecule metabolic process | GO:0060255 | 45 | 0.000146 | 0.00754 |
| nitrogen compound metabolic process | GO:0006807 | 52 | 0.000148 | 0.007544 |
| cellular protein metabolic process | GO:0044267 | 42 | 0.000187 | 0.009326 |
| single-organism transport | GO:0044765 | 25 | 0.000217 | 0.010622 |
| intracellular organelle lumen | GO:0070013 | 32 | 0.000243 | 0.011321 |
| membrane-enclosed lumen | GO:0031974 | 32 | 0.000243 | 0.011321 |
| organelle lumen | GO:0043233 | 32 | 0.000243 | 0.011321 |
| negative regulation of macromolecule metabolic process | GO:0010605 | 23 | 0.000249 | 0.011402 |
| nucleic acid metabolic process | GO:0090304 | 40 | 0.000265 | 0.01182 |
| biosynthetic process | GO:0009058 | 47 | 0.000271 | 0.01182 |
| response to stimulus | GO:0050896 | 55 | 0.000274 | 0.01182 |
| chromosome | GO:0005694 | 14 | 0.000276 | 0.01182 |
| transition metal ion binding | GO:0046914 | 18 | 0.000281 | 0.011855 |
| negative regulation of cellular process | GO:0048523 | 35 | 0.00029 | 0.012056 |
| organelle fusion | GO:0048284 | 6 | 0.000306 | 0.012334 |
| single-organism membrane fusion | GO:0044801 | 6 | 0.000306 | 0.012334 |
| mitochondrion | GO:0005739 | 20 | 0.000337 | 0.013408 |
| fat cell differentiation | GO:0045444 | 6 | 0.000376 | 0.014642 |
| cellular biosynthetic process | GO:0044249 | 45 | 0.000379 | 0.014642 |
| single-organism developmental process | GO:0044767 | 43 | 0.000408 | 0.015565 |
| lysosome | GO:0005764 | 8 | 0.000438 | 0.016243 |
| lytic vacuole | GO:0000323 | 8 | 0.000438 | 0.016243 |
| chromosomal part | GO:0044427 | 13 | 0.000465 | 0.01701 |
| developmental process | GO:0032502 | 43 | 0.0005 | 0.018049 |
| catalytic activity | GO:0003824 | 51 | 0.00054 | 0.019225 |
| vesicle organization | GO:0016050 | 7 | 0.000589 | 0.020708 |
| negative regulation of cellular metabolic process | GO:0031324 | 22 | 0.000613 | 0.021278 |
| cellular component assembly | GO:0022607 | 24 | 0.000628 | 0.021291 |
| establishment of localization in cell | GO:0051649 | 18 | 0.000629 | 0.021291 |
| microtubule-based process | GO:0007017 | 10 | 0.000652 | 0.02183 |
| regulation of cell cycle | GO:0051726 | 12 | 0.000672 | 0.022014 |
| vesicle fusion | GO:0006906 | 5 | 0.000674 | 0.022014 |
| vesicle-mediated transport | GO:0016192 | 14 | 0.000705 | 0.022741 |
| regulation of localization | GO:0032879 | 21 | 0.000736 | 0.023485 |
| cellular component organization | GO:0016043 | 44 | 0.000821 | 0.025607 |
| cell proliferation | GO:0008283 | 18 | 0.000821 | 0.025607 |
| nucleoplasm | GO:0005654 | 22 | 0.00086 | 0.026496 |
| regulation of biosynthetic process | GO:0009889 | 32 | 0.000936 | 0.027435 |
| protein modification process | GO:0036211 | 32 | 0.000936 | 0.027435 |
| cellular protein modification process | GO:0006464 | 32 | 0.000936 | 0.027435 |
| membrane fusion | GO:0061025 | 6 | 0.000942 | 0.027435 |
| organelle membrane fusion | GO:0090174 | 5 | 0.000944 | 0.027435 |
| response to stress | GO:0006950 | 26 | 0.000957 | 0.027435 |
| microtubule cytoskeleton organization | GO:0000226 | 8 | 0.000978 | 0.027435 |
| multicellular organismal reproductive process | GO:0048609 | 8 | 0.000978 | 0.027435 |
| organic substance transport | GO:0071702 | 22 | 0.001001 | 0.027435 |
| macromolecular complex | GO:0032991 | 42 | 0.001013 | 0.027435 |
| cellular localization | GO:0051641 | 21 | 0.001014 | 0.027435 |
| macromolecule modification | GO:0043412 | 33 | 0.001026 | 0.027435 |
| cellular component organization or biogenesis | GO:0071840 | 45 | 0.001029 | 0.027435 |
| multicellular organism reproduction | GO:0032504 | 8 | 0.001035 | 0.027435 |
| organic substance biosynthetic process | GO:1901576 | 44 | 0.001067 | 0.027485 |
| phosphoric diester hydrolase activity | GO:0008081 | 4 | 0.001067 | 0.027485 |
| protein complex localization | GO:0031503 | 4 | 0.001067 | 0.027485 |
| cellular component biogenesis | GO:0044085 | 25 | 0.001142 | 0.029138 |
| nuclear chromosome part | GO:0044454 | 9 | 0.001155 | 0.029206 |
| cilium morphogenesis | GO:0060271 | 6 | 0.001199 | 0.030046 |
| cellular nitrogen compound metabolic process | GO:0034641 | 46 | 0.001211 | 0.030075 |
| nuclear chromosome | GO:0000228 | 9 | 0.001397 | 0.034367 |
| intracellular non-membrane-bounded organelle | GO:0043232 | 33 | 0.001422 | 0.034367 |
| non-membrane-bounded organelle | GO:0043228 | 33 | 0.001422 | 0.034367 |
| protein complex | GO:0043234 | 36 | 0.001448 | 0.034692 |
| regulation of cellular biosynthetic process | GO:0031326 | 31 | 0.00157 | 0.037038 |
| MAPK cascade | GO:0000165 | 10 | 0.00158 | 0.037038 |
| zinc ion binding | GO:0008270 | 14 | 0.0016 | 0.037038 |
| Golgi apparatus | GO:0005794 | 14 | 0.0016 | 0.037038 |
| cellular developmental process | GO:0048869 | 31 | 0.001698 | 0.038976 |
| G2/M transition of mitotic cell cycle | GO:0000086 | 4 | 0.00185 | 0.042112 |
| single organism reproductive process | GO:0044702 | 10 | 0.002087 | 0.046723 |
| DNA damage checkpoint | GO:0000077 | 4 | 0.002094 | 0.046723 |
| heterocycle biosynthetic process | GO:0018130 | 31 | 0.002103 | 0.046723 |
| regulation of response to stimulus | GO:0048583 | 26 | 0.002192 | 0.048099 |
| regulation of gene expression | GO:0010468 | 31 | 0.0022 | 0.048099 |
| chromosomal region | GO:0098687 | 7 | 0.002278 | 0.049413 |
